# Peroxisome proliferator-activated receptor gamma gene variants modify human airway and systemic responses to indoor dibutyl phthalate exposure

**DOI:** 10.1186/s12931-022-02174-8

**Published:** 2022-09-16

**Authors:** Clarus Leung, Min Hyung Ryu, Anette Kocbach Bølling, Danay Maestre-Batlle, Christopher F. Rider, Anke Hüls, Oscar Urtatiz, Julie L. MacIsaac, Kevin Soon-Keen Lau, David Tse Shen Lin, Michael S. Kobor, Chris Carlsten

**Affiliations:** 1 Department of Medicine, University of British Columbia, 7th Floor, 2775 Laurel St, VancouverVancouver, BC V5Z1M9 Canada; 2 Department of Air Pollution and Noise, Norwegian Institute of Public Health, Oslo, Norway; 3 Department of Epidemiology and Gangarosa Department of Environmental Health, Rollins School of Public Health, Emory University, Atlanta, GA USA; 4 Department of Medical Genetics, University of British Columbia-BC Children’s Hospital Research Institute, Vancouver, BC Canada

**Keywords:** Airway inflammation, Allergen challenge, Peroxisome proliferator-activated receptor gamma, Phthalates, Polymorphism, PPAR-gamma

## Abstract

**Background:**

Single nucleotide polymorphisms (SNPs) of peroxisome proliferator-activated receptor gamma (PPAR-γ; gene: *PPARG)* and oxidative stress genes are associated with asthma risk. However, whether such variants modulate responses to dibutyl phthalate (DBP), a common plasticizer associated with increased asthma development, remains unknown. The purpose of this study is to investigate how SNPs in *PPARG* and oxidative stress genes*,* as represented by two separate genetic risk scores, modify the impact of DBP exposure on lung function and the airway and systemic response after an inhaled allergen challenge.

**Methods:**

We conducted a double-blinded human crossover study with sixteen allergen-sensitized participants exposed for three hours to DBP and control air on distinct occasions separated by a 4-week washout. Each exposure was followed by an allergen inhalation challenge; subsequently, lung function was measured, and blood and bronchoalveolar lavage (BAL) were collected and analyzed for cell counts and allergen-specific immunoglobulin E (IgE). Genetic risk scores for PPAR-γ (P-GRS; weighted sum of *PPARG* SNPs rs10865710, rs709158, and rs3856806) and oxidative stress (OS-GRS; unweighted sum of 16 SNPs across multiple genes) were developed, and their ability to modify DBP effects were assessed using linear mixed-effects models.

**Results:**

P-GRS and OS-GRS modified DBP effects on allergen-specific IgE in blood at 20 h (interaction effect [95% CI]: 1.43 [1.13 to 1.80], p = 0.005) and 3 h (0.99 [0.98 to 1], p = 0.03), respectively. P-GRS also modified DBP effects on Th2 cells in blood at 3 h (− 25.2 [− 47.7 to − 2.70], p = 0.03) and 20 h (− 39.1 [− 57.9 to − 20.3], p = 0.0005), and Th2 cells in BAL at 24 h (− 4.99 [− 8.97 to − 1.01], p = 0.02). An increasing P-GRS associated with reduced DBP effect on Th2 cells. Neither GRS significantly modified DBP effects on lung function parameters.

**Conclusions:**

PPAR-γ variants modulated several airway and systemic immune responses to the ubiquitous chemical plasticizer DBP. Our results suggest that PPAR-γ variants may play a greater role than those in oxidative stress-related genes in airway allergic responses to DBP.

*Trial registration:* This study reports results from The Phthalate-Allergen Immune Response Study that was registered on ClinicalTrials.gov with identification NCT02688478.

**Supplementary Information:**

The online version contains supplementary material available at 10.1186/s12931-022-02174-8.

## Background

Peroxisome proliferator-activated receptors (PPAR) are ligand-activated transcription factors expressed in the airway epithelium, airway smooth muscle cells, alveolar macrophages, T lymphocytes, and eosinophils [1–3]. Notably, PPAR-γ (NR1C3) regulates systemic and airway allergic immune responses relevant in asthma [4, 5].

Genetic variants or single nucleotide polymorphisms (SNPs) in *PPARG* and oxidative stress-related genes have been associated with increased risk of asthma development and exacerbations [6–11], highlighting gene-environment interactions in asthma pathophysiology. In one study of Koreans, the *PPARG* polymorphism rs3856806 was associated with asthma development [7]. However, Palmer et al*.* reported that rare alleles of *PPARG* (rs1805192 and rs3856806) were associated with a reduced number of asthma exacerbations in a Caucasian population [8]. In a case–control study by Li et al., asthma risk was influenced by the rs1805192 and rs10865710 *PPARG* polymorphisms in a Chinese population [6]. Variants of the oxidative stress-related genes are associated with phthalate urine concentrations and modified the association of phthalate exposure with asthma in children [12]. In a group of Korean elders, variants of certain oxidative stress-related genes influenced the association between urinary phthalate levels and decreasing lung function [13].

Phthalates are suspected to interact with PPAR-γ receptors [14]. Phthalates are synthetic diesters of phthalic acid (1,2-benzenedicarboxylic acid) used to enhance properties of plastics such as flexibility, transparency, and durability [14]. Humans are exposed to these environmental contaminants through a wide variety of common consumer products, such as household furnishings, food packaging, and cosmetics [15, 16]. Phthalate exposure has been associated with allergic diseases in epidemiological studies [16]. In animal models, inhalation toxicity and oral intake of phthalates have demonstrated increased airway inflammation following exposures [16]. In vitro studies to date indicate that exposure to environmentally relevant concentrations of certain phthalates can increase the release of inflammatory mediators from macrophages and lymphocytes, decrease phagocytic activity, and modulate differentiation of dendritic cells [17, 18].

We have previously published results from this randomized, crossover human exposure study which demonstrated that acute indoor exposure to dibutyl phthalate (DBP) enhanced the allergen-associated drop in forced expiratory volume in 1 s (FEV_1_) [19]. DBP and allergen co-exposure significantly increased the percentage of M2 macrophages and macrophage activation markers in BAL, but had modest effects on airway immune mediators [19].

This novel study explores whether the effects of DBP on lung function, immune cells, and allergen-specific IgE are associated with SNPs related to *PPARG* (P-GRS) or oxidative stress-related genes (OS-GRS), as represented by genetic risk scores. We hypothesized that both the P-GRS and OS-GRS modulate the immune response to DBP, with higher scores associated with larger allergen-driven immune responses.

## Methods

The study was registered on ClinicalTrials.gov with identification NCT02688478 and was approved by the research ethics board at the University of British Columbia (H14-01119) and the Norwegian Regional Committees for Medical and Health Research Ethics (2014/1217).

### Participants and study design

Sixteen allergen-sensitized, non-smoking participants between 19–49 years of age were enrolled in this double-blinded, order-randomized, crossover study [19]. The individuals were either healthy or had mild asthma (FEV_1_ greater than 70%); and were allergen-sensitized to grass, birch, or house dust mite. Airway hyperresponsiveness (AHR) status was categorized as either hyperresponsive (provocative concentration of methacholine resulting in 20% drop in FEV_1_ [PC20] ≤ 16 mg/ml) or normally responsive (PC20 > 16 mg/ml). Participants with asthma taking oral or inhaled corticosteroids were excluded. All participants discontinued their bronchodilator, antihistamine, and nonsteroidal anti-inflammatory medications 7 days prior to each exposure and during exposure days, except for salbutamol (200 µg was given routinely after methacholine challenge [by protocol, 20 h after exposure]).

Participants were randomized to the order of 3-h indoor air exposures to DBP (nominal concentration of 150 µg/m^3^) and control air (CA) at the Air Pollution Exposure Laboratory, University of British Columbia. Exposures were separated by at least 4 weeks for washout. Full details of the exposure methods were previously reported [19].

### Outcome measures and analysis

**Fig. 1:**
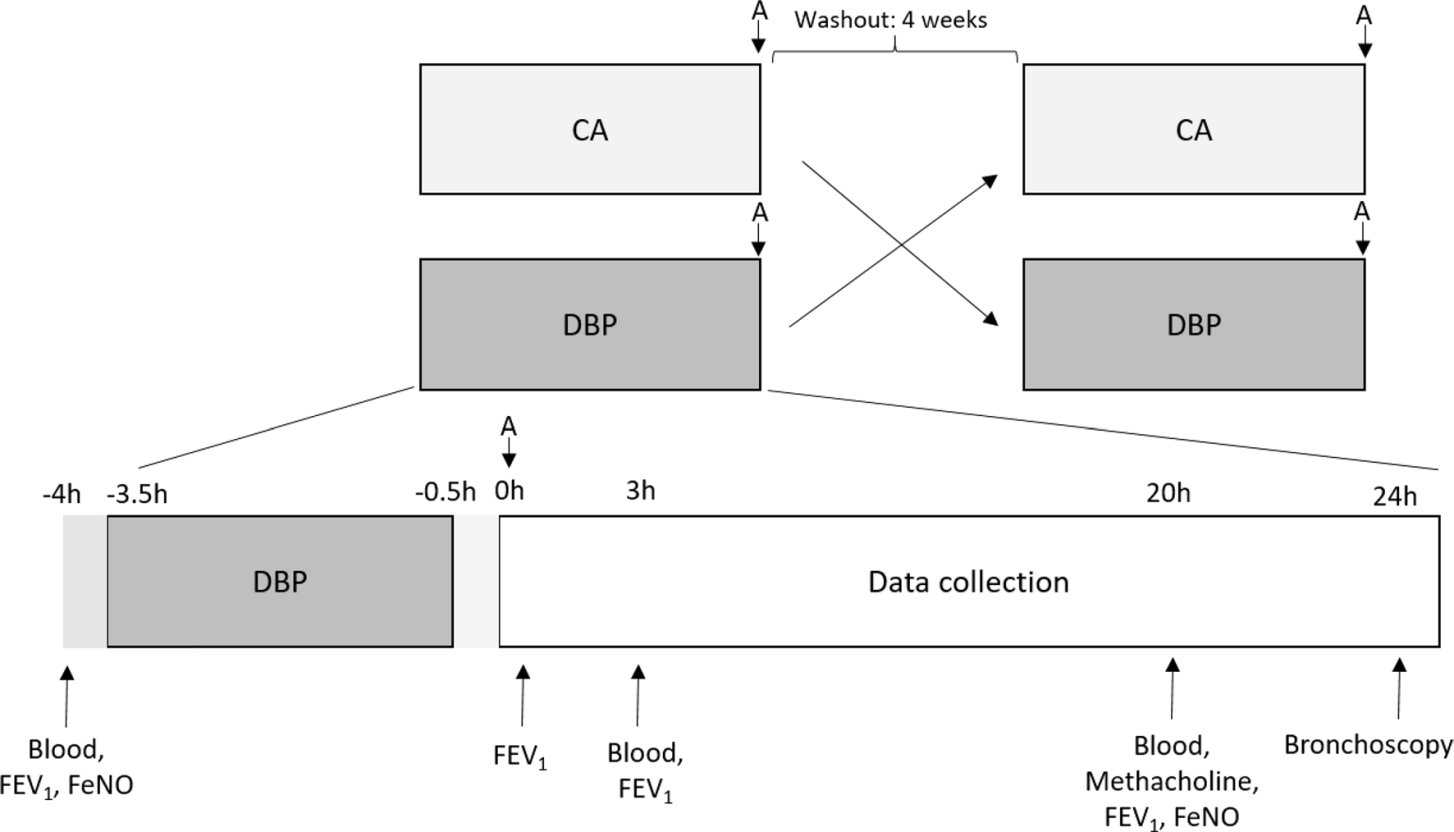
Study design and data collection timepoints. A double-blinded, placebo-controlled, human exposure study to dibutyl phthalate (DBP) was performed. Research participants underwent allergen inhalation challenge (A = grass, house dust mite, or birch) following 3-h exposure to control air (CA) or DBP. Airflow limitation (FEV_1_) was monitored from baseline to 20 h post allergen challenge. Blood was collected at − 4 h (baseline), 3 h, and 20 h; and bronchoscopies were performed at 24 h after the first exposure pre-washout and after the second exposure post-washout. Allergen-specific IgE and immune cell proportions were assayed in blood and bronchoalveolar lavage

The study design and data collection time are outlined in Fig. 1. Data were collected after each exposure (DBP and CA) in each crossover study arm. Blood samples were collected at − 4 h (baseline), 3 and 20 h after the allergen challenge post-DBP or CA exposure, while BAL was collected at 24 h after the allergen challenge post-DBP or CA exposure via bronchoscopy. These samples were analyzed for allergen-specific IgE, and blood and airway immune cells. The allergen-specific IgE level in blood and BAL was determined by ImmunoCAP Specific IgE analysis on a Phadia 250 system (Thermo Fisher Scientific). Immunophenotyping was performed by flow cytometry with analysis completed in FCS Express (v6.04.0034; De Novo Software). Further details are provided in the Additional file 1. The DBP effects on immune cells, which reflect primary endpoints rather than gene-exposure analyses, were previously reported [19].

FEV_1_ was measured at 3 and 20 h after exposure; while methacholine challenge and fractional exhaled nitric oxide (FeNO) were measured at 20 h after exposure. Delta values were calculated for each FEV_1_ timepoint and FeNO by subtracting the baseline values measured at -4 h. Allergen area under the curve (AUC) was determined as the percent fall in FEV_1_ spanning baseline to 3 h after inhaled allergen challenge.

### Genotyping

Genotyping was performed on the DNA extracted from peripheral blood mononuclear cells (PBMC). Details of the genotyping are presented in the Additional file 1. The DNA sequences used to perform targeted pyrosequencing are listed in Additional file 1: Table S1.

### Genetic risk scores

The GRS was developed from 3 SNPs of *PPARG* (P-GRS) that have been associated with an increased asthma risk and have minor allele frequency greater than 1% [6, 8]. The details, chromosome location, and risk alleles of the SNPs are illustrated in Table 1. The P-GRS was weighted as the sum of the effect estimates of the 3 SNPs: rs10865710, rs709158, and rs3856806 [6]. The genotypes and corresponding weights for each SNP of the *PPAR-γ* GRS (P-GRS) are displayed in Additional file 1: Tables S1, S2. Genotyping was also performed for a 4^th^ SNP of *PPARG*, rs1805192. This SNP did not vary among the participants, as shown in Additional file 1: Table S3. This SNP was therefore not included in the GRS.

**Table 1:** Characteristics of the selected *PPARG* single nucleotide polymorphisms used to generate P-GRS

SNP	Chromosome	Other names	Functional/structural change	Risk allele
rs10865710	3:12311699 (GRCh38) 3:12353198 (GRCh37)	C-681G	Intron* C > G, Upstream transcript variant	C
rs709158	3:12421677 (GRCh38) 3:12463176 (GRCh37)	Intron A > G	Intron* A > G, Genic downstream transcript variant	A
rs3856806	3:12,434,058 (GRCh38) 3:12,475,557 (GRCh37)	C1341T, His449His / His447His	3 prime UTR variant/synonymous variant	C

*SNP*  single nucleotide polymorphism

Our analyses also included an oxidative stress genetic risk score (OS-GRS) based on 16 allelic variants that have been suggested to modulate the response to air pollution and tobacco smoke via oxidative stress pathway [20–27], Additional file 1: Table S4. Selected genes were derived from a number of individual studies with heterogeneous endpoints, thus, no adequate external weights of the relative contribution of each variant were available for the development of weighted risk scores in OS-GRS [28].

### Statistical analysis

Statistical tests were performed using linear mixed-effects (LME) models (nlme package version 3.1-151) in R, version 4.0.3 (R Foundation for Statistical Computing) implemented in R Studio, version 1.3.1093 (RStudio). Confidence intervals (CI) were computed using the gmodels package (version 2.18.1) in RStudio. Interaction plots for study endpoints were generated using R packages: LME4 (version 1.1-27) and interplot (version 0.2.3). All LME analyses for analytes in blood were performed for delta values, calculated for 3 h and 20 h timepoints relative to the baseline (− 4 h), for each individual and each exposure condition. When necessary, data were log_10_-transformed to normalize the data distribution. Any effect estimates and associated confidence intervals are based on log_10_-transformed data.

In our primary LME model for allergen-specific IgE level, exposure (DBP relative to CA) was the fixed effect, and participant ID was set as a random effect. To assess whether either of the two GRS modified the effect of DBP on the study outcomes, a model with the interaction term exposure-by-GRS (exposure*GRS) as a fixed effect was evaluated. Participant ID was set as a random effect in these models. P-values < 0.05 were considered statistically significant.

## Results

Ten females and 6 males enrolled in the PAIR study were included in these analyses. Eleven out of the 16 participants underwent research bronchoscopies. Baseline participant profiles at screening, FEV_1_ measurements, and P-GRS are shown in Table 2. Eight participants were categorized as hyperresponsive AHR status based on baseline methacholine PC_20_. The mean P-GRS among participants was − 0.12 ± 0.15, with a more negative number indicating a lower risk score and a more positive number (maximum is 0) indicating a higher risk score, i.e. greater risk of having asthma.

**Table 2:** Participant characteristics and P-GRS profiles

Participant	Sex	Age	Baseline FEV_1_ (% predicted)	Baseline methacholine PC_20_ (mg/ml)	P-GRS
1	F	23	76	0.3	− 0.02
2	F	33	88	1.7	0
3	M	29	96	2	− 0.36
4	F	46	79	2.9	0
5	F	45	102	6.9	− 0.05
6	F	45	N/A*	9.1	− 0.05
7	M	36	99	14.5	− 0.38
8	M	27	93	16	− 0.02
9	F	21	95	47.9	0
10	F	34	91	64	− 0.09
11	F	21	99	121.1	− 0.05
12	M	26	94	147.1	0
13	F	26	108	149.3	− 0.38
14	F	29	117	301	− 0.05
15	M	31	74	491.5	− 0.36
16	M	26	101	1067	− 0.05
Summary	M = 6 F = 10	31 ± 8^#^	94 ± 12^#^	AHR = 8†	− 0.12 ± 0.15^#^

*N/A*  not available, *FEV*_*1*_  forced expiratory volume in 1 s, *PC*_*20*_  provocative concentration of methacholine causing a 20% drop in FEV_1_, *P-GRS*  *PPARG* genetic risk score, calculated as the weighted additive effects of minor alleles of 3 selected SNPs. ^#^Mean ± SD. *Baseline spirometry not available for this participant. Baseline lung function measurements were taken at the time of study recruitment. †Airway hyperresponsiveness (AHR) status defined as provocative concentration of methacholine resulting in 20% drop in FEV1 [PC20] ≤ 16 mg/ml

**Fig. 2:**
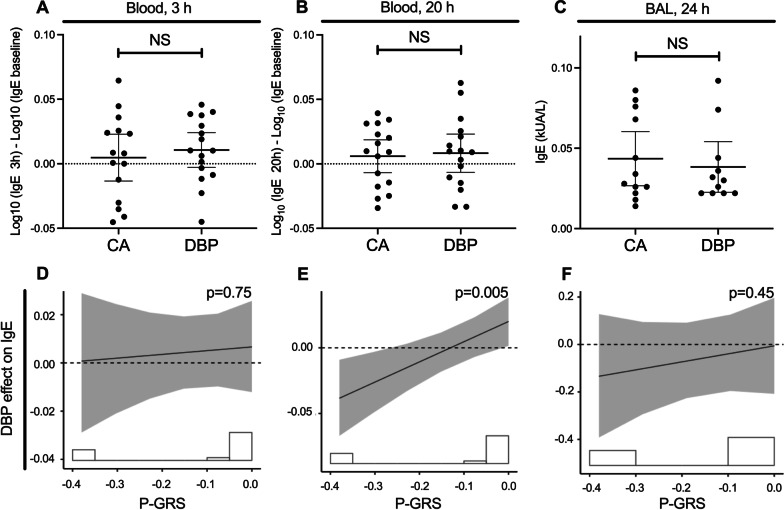
DBP effect for allergen-specific IgE level and the interaction with P-GRS. Blood allergen-specific IgE after the allergen challenge post-DBP compared to control at 3 and 20 h (**A**, **B**), and BAL allergen-specific IgE at 24 h (**C**) are shown. The interaction by P-GRS on DBP effect at respective time points is shown in **D**–**F**. Blood and BAL samples were collected from 16 and 11 participants, respectively. The histogram underlying each plot illustrates the distribution of the participants’ P-GRS. Effect values represent the DBP-attributable difference between the measurement at the given time point and the baseline. The solid line represents the interaction of P-GRS on allergen-specific IgE due to DBP compared to CA, with the p value corresponding to the interaction significance. The shaded region represents 95% confidence intervals

The 3-h exposure to DBP did not significantly change allergen-specific IgE levels compared to CA in blood (3 h and 20 h) or BAL (24 h) (Fig. 2A–C). However, there was a significant exposure-by-P-GRS interaction effect at 20 h (interaction effect [95% CI]: 1.43 [1.13 to 1.80], p = 0.005). The positive slope of the interaction line indicates that increasing P-GRS was associated with increasing DBP effect on allergen-specific IgE in blood (Fig. 2E). Participants with a higher P-GRS experienced a larger DBP effect on IgE at 20 h compared to baseline. There was no significant interaction of P-GRS at 3 h and 24 h in blood and BAL, respectively (Fig. 2D, F).

**Fig. 3:**
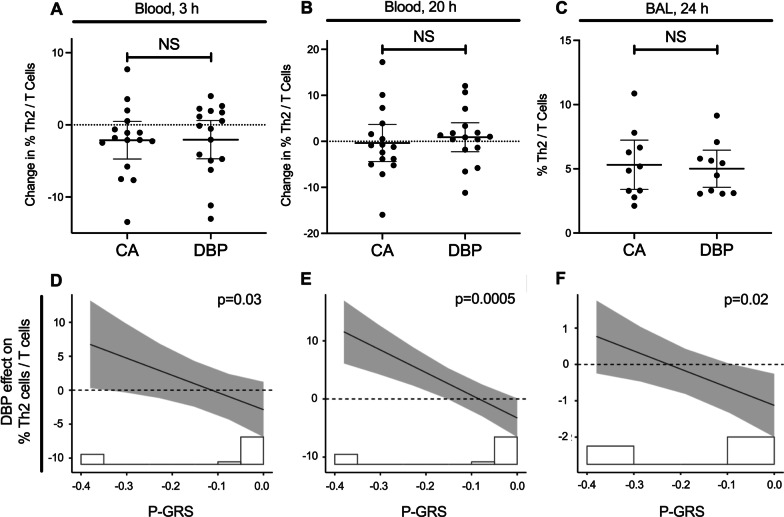
DBP effect for Th2 lymphocytes and the interaction with P-GRS. Blood % Th-2 lymphocytes in T-cells after the allergen challenge post-DBP compared to control at 3 and 20 h (**A**, **B**), and BAL % Th2 at 24 h (**C**) are shown. The interaction by P-GRS on DBP effect at respective time points is shown in **D**–**F**. Blood and BAL samples were collected from 16 and 11 participants, respectively. The histogram underlying each plot illustrates the distribution of the participants’ P-GRS. Effect values represent the DBP-attributable difference between the measurement at the given time point and the baseline. The solid line represents the interaction of P-GRS on Th2 lymphocytes due to DBP compared to CA, with the p value corresponding to the interaction significance. The shaded region represents 95% confidence intervals

DBP exposure did not significantly affect Th2 lymphocytes percentages compared to CA in the blood (3 h and 20 h) or BAL (24 h) (Fig. 3A–C). The DBP effect on % Th2 lymphocytes was modified by P-GRS, such that those participants with higher P-GRS were associated with decreasing DBP effect on % Th2 (Fig. 3D–F). This was observed for % Th2 in blood at 3 and 20 h (interaction effect [95% CI]: − 25.2 [− 47.7 to − 2.70], p = 0.03; and − 39.1 [− 57.9 to − 20.3], p = 0.0005), and in BAL at 24 h (− 4.99 [− 8.97 to − 1.01], p = 0.02). Participants with a higher P-GRS experienced a smaller DBP effect on Th2 lymphocytes at 3 h, 20 h, and 24 h compared to baseline. There was however no significant exposure-by-P-GRS interaction for other immune cells in blood or BAL, as shown in Additional file 1: Table S5.

**Fig. 4:**
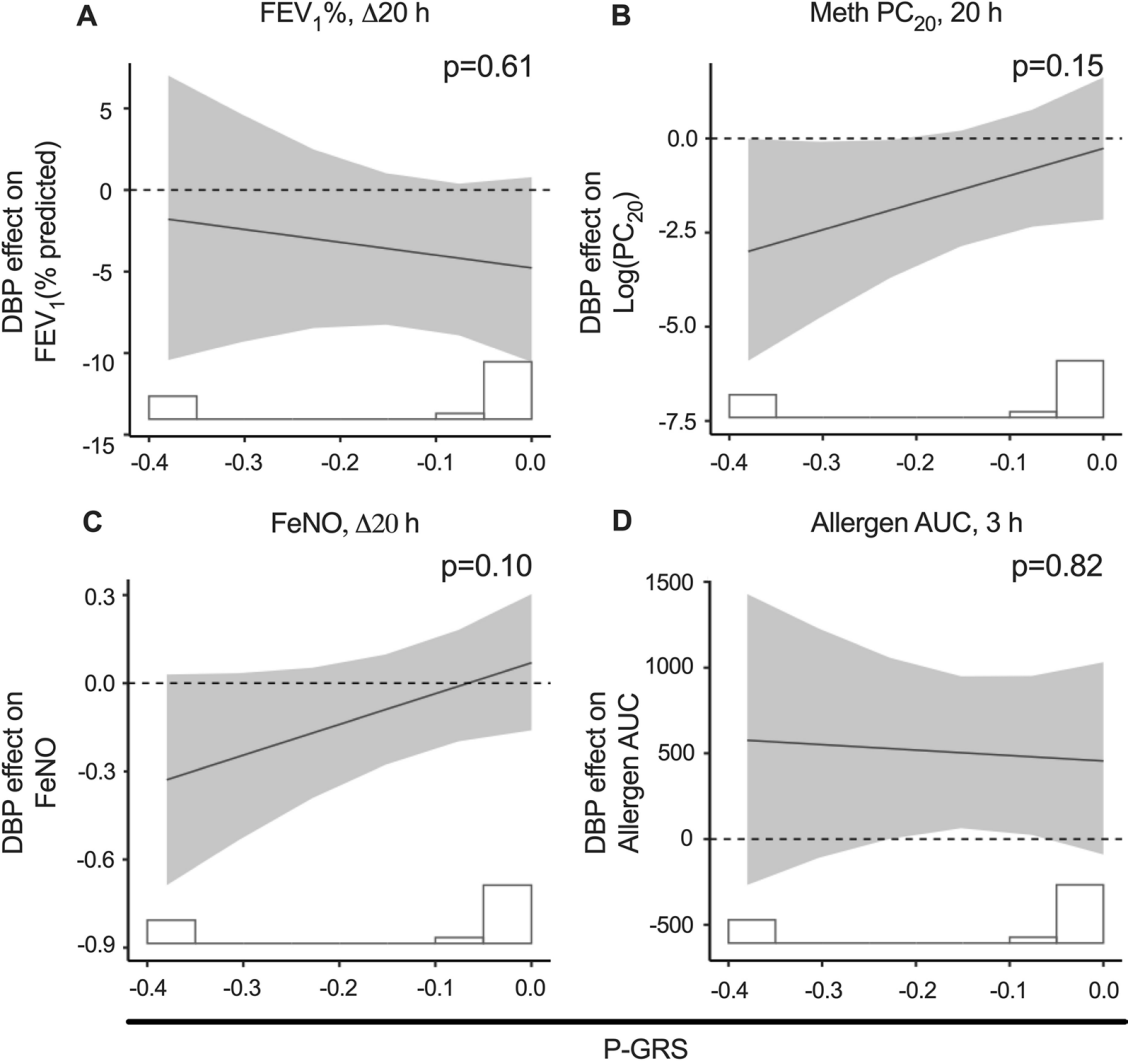
Exposure-by-P-GRS interaction on DBP effect on lung function outcomes. Lung function outcomes included: **A** FEV_1_ (% predicted), ∆20 h; **B** Methacholine challenge (LogPC_20_), 20 h; **C** FeNO, ∆20 h; **D** Allergen AUC, 3 h. The listed time points refer to the time after the allergen challenge post-DBP or CA exposure. *FEV*_*1*_  forced expiratory volume in one second, *PC*_*20*_ concentration leading to 20% fall in FEV_1_, *FeNO*  fractional exhaled nitric oxide, *AUC*  area under curve, measured as percent decline in FEV_1_ spanning baseline to 3 h after allergen challenge. The histogram underlying each plot illustrates the distribution of the participants’ P-GRS. Effect values represent the DBP-attributable difference between the measurement at the given time point and the baseline. The solid line represents the interaction effect, with the p value corresponding to the interaction significance. The shaded region represents 95% confidence intervals

Lung function outcomes included allergen AUC (from baseline to 3 h post allergen challenge), FEV_1_ and FeNO (∆20 h), and methacholine challenge (20 h). The DBP effect on these outcomes was not significantly modified by P-GRS (Fig. 4).

The unweighted OS-GRS composed of 16 genes for each participant are listed in Additional file 1: Table S6. The mean OS-GRS was 9.7 ± 2.6, with a more positive number indicating a higher risk score (theoretical maximum is 32). There was a significant exposure-by-OS-GRS interaction (interaction effect [95% CI]: 0.99 [0.98 to 1], p = 0.03) such that increasing OS-GRS was associated with decreasing DBP effect on blood allergen-specific IgE at 3 h (Additional file 1: Fig. S1A). There was no significant interaction of OS-GRS for DBP effect on allergen-specific IgE and % Th2 lymphocytes at other time points (Additional file 1: Fig. S1B–F). OS-GRS did not modify the DBP effect on lung function parameters (Additional file 1: Fig. S2).

## Discussion

This study demonstrated that a genetic risk score based on three relevant *PPARG* polymorphisms may modify the DBP effect on two important mediators of allergic responses, allergen-specific IgE levels and the proportion of Th2 cells amongst lymphocytes. None of these endpoints were affected by the DBP exposure alone. In contrast to P-GRS, the impact of OS-GRS was limited. Thus, our findings suggest that PPAR-γ may play a role in the airway allergic effects in response to DBP, while oxidative stress does not appear to be substantially involved.

IgE plays an important role in the acute airway allergic response to allergens, by binding to mast cells and basophils to release histamine, neutral proteases, and chemotactic factors [29]. IgE and Th2 lymphocytes are relevant in the DBP-related airway inflammation, and several phthalates have been shown to increase the release of Th2-associated mediators and modulates Th1/Th2 balance [16]. We observed that individuals with a higher P-GRS augmented the DBP effect on blood allergen-specific IgE at 20 h. The interaction of P-GRS with DBP-induced effects on IgE level was opposite to that of Th2 lymphocytes. Moreover, the DBP effect on B cells, which are the main cells that produce IgE, were not significantly modified by P-GRS. In a prior study where a bronchoscopic segmental allergen challenge was performed in atopic asthmatic subjects, there was a significant increase in allergen-specific IgE in the bronchoalveolar lavage fluid 24 h after the challenge [30]. In contrast, the observed rises in specific IgE in the serum was much smaller, suggesting a predominantly local allergen response that is independent of systemic alterations [30]. Furthermore, the production of IgE after an inhaled allergen challenge appears to be mediated through local expansion of IgE^+^ B cells in the airways rather than in the blood, with the latter being a more delayed response [31]. Overall, the interaction of P-GRS with DBP-related Th2 lymphocyte response in the blood and airways was more uniform and substantial compared to the observation regarding blood allergen-specific IgE alone.

Given these observations, the overall trend is a reduction in DBP-induced effects for increasing P-GRS, or higher asthma risk [6]. A possible interpretation is that individuals with a low P-GRS are more susceptible to DBP-induced effects in the airways. This is somewhat analogous with the main result in the previous PAIR study which demonstrated that airway responsiveness increased by 48% after DBP exposure specifically in participants without baseline hyperresponsiveness, exacerbating the mean response to levels typical of those with airway hyperresponsiveness [19].

In our report of primary endpoints in this study, we showed that DBP exposure augmented the allergen-induced recruitment of total macrophages in the airways and there was skewing towards M2 macrophage phenotype in the BAL [19]. However, we did not find an exposure-by-P-GRS interaction on the DBP effect for macrophages in the current analysis, suggesting that augmented macrophages recruitment in the lung may not be impacted by the genetic variation in PPAR-γ. Furthermore, Maestre-Batlle et al. reported that exposure to DBP resulted in significant augmentation of allergen-induced AHR as measured by greater decrease methacholine PC20 and FEV_1_ change in participants without AHR [19]. But, the present study did not find a significant exposure-by-P-GRS interaction on lung function. Impact on the lung function likely reflects cumulative immune responses in the airways which modulate airway smooth muscle function rather than reflecting modulation of Th2 cells or IgE production. Our data suggests that genetic variation in PPAR-γ only impacted IgE and Th2 cells.

Asthma is a complex disease with numerous genetic determinants. The weighted GRS is an intuitive approach for combining genetic data into a single measure of susceptibility to a disease or physiological response [32]. The weighted score was preferred over a simple-count genetic score equal to the sum of its risk alleles because the SNPs used in our study were derived from a prior observational study with reported odds ratio and effect estimates, allowing us to approximate the relative weights of each SNP [6]. However, we acknowledge that our estimate of genetic risk is still crude as it contains only three susceptibility polymorphisms that are associated with asthma. In our study, two of the three SNPs, rs10865710 and rs709158 are intron variants of the PPAR-γ gene [33, 34]. We found that polymorphism in rs10865710 alone was sufficient to show effect modification on Th2 lymphocytes and allergen-specific IgE levels in blood and BAL (Additional file 1: Fig. S1). We also note a bimodal distribution of P-GRS. This is due to the weighing of different SNPs based on asthma odds ratio reported in previous research [6]. Specifically, rs10865710 G/G genotype conferred the greatest reduction in risk (odd ratio (OR) = 0.53) for asthma compared to rs709158 GG (OR = 0.91) and rs3856806 (OR = 0.97). Since those individuals with rs10865710 G/G genotype (n = 4) had the smallest OR and consequently, a much lower P-GRS than the other genotypes, this resulted in a bimodal distribution of P-GRS. It is unknown if the selected SNPs in this study, though associated with an important clinical outcome, are involved with the interaction of phthalates and the ligand-binding sites on PPAR-γ receptors.

Although previous studies suggest that even a single SNP can mediate a profound effect on the regulation of phenotype [34], the optimal number of SNPs to utilize in creating a weighted GRS is still under debate. The strategy used to construct a GRS, whether based on the protein biological function or structure, is also contested. In our case, another approach to create the GRS would be to select SNPs specifically involved in the receptor ligand-binding domain through which phthalates are thought to interact. Thus, our study only suggests a plausible, albeit indirect relationship between these polymorphisms and PPAR-γ function. Further studies with SNPs relevant to the ligand-binding domain should be performed to assess whether PPAR-γ receptors play a role in the molecular cascade initiated by DBP in human airway cells. Larger studies are required for validating the clinical relevance of PPAR-γ polymorphisms.

The current findings of effect modification of PPAR-γ polymorphisms on DBP induced effects should be used to focus future experimental efforts. The modest participant sample size and the weighted P-GRS may have limited variations in the GRS among the participants as evident in the P-GRS distribution. Moreover, the BAL and blood samples collected in this study only captured cellular and clinical outcomes at defined time points within 24 h post-allergen and DBP exposure, and the long-term risk of allergic and inflammatory airway responses is unknown. Finally, OS-GRS had a limited impact on DBP-induced effects, which may reflect differences in study design and endpoints, and phthalate type and concentration compared to other studies.

## Conclusions

Several genetic variations in *PPARG,* as represented by a genetic risk score, modified the impact of dibutyl phthalate exposure on allergen-specific IgE and Th2 lymphocytes after allergen challenge in a controlled human exposure study. Our study focused specifically on DBP given its real-world propensity for exposure by inhalation, and our findings may motivate further study for potential application to personal and public health practice and policy.

## Supplementary Information

**Additional file 1: Table S1.** DNA sequences for PPARG used to perform targeted pyrosequencing. **Table S2.** The genotypes and GRS are illustrated for rs709158, rs3856806, and rs10865710. **Table S3.** The weights of each SNP in the construction of P-GRS. **Table S4.** Oxidative stress-related genes included in the associated OS-GRS and definition of their risk alleles. **Table S5.** Exposure-by-P-GRS interaction on DBP effects on (A) blood immune cells at 3h, (B) blood immune cells at 20h, and (C) BAL immune cells at 24h. **Table S6.** Participant characteristics and oxidative stress genetic risk score (OS-GRS). **Figure S1.** Effect of OS-GRS interaction on DBP effect on IgE and Th2 lymphocytes. Blood allergen-specific IgE at 3 and 20 h (A-B), and BAL allergen-specific IgE at 24 h (C); blood % Th2 lymphocytes at 3 and 20 h (D-E), and BAL % Th2 at 24 h (F) after the allergen challenge post-DBP. The histogram underlying the plot illustrates the distribution of the participants’ OS-GRS. Blood and BAL samples were collected for 16 and 11 participants, respectively. The histogram underlying each plot illustrates the distribution of the participants’ OS-GRS. Effect values represent the DBP-attributable difference between the measurement at the given time point and the baseline. The solid line represents the interaction effect, with the p value corresponding to the interaction significance. The shaded region represents 95% confidence intervals. **Figure S2.** Exposure-by-OS-GRS interaction on DBP effect on lung function outcomes. Lung function outcomes included: (A) FEV_1_ (% predicted), ∆20 h; (B) Methacholine challenge (LogPC20), 20 h; (C) FeNO, ∆20 h; (D) Allergen AUC, 3 h. The listed time points refer to the time after the allergen challenge post-DBP or CA exposure. FEV_1_ = forced expiratory volume in one second; PC20 = concentration leading to 20% fall in FEV_1_; FeNO = fractional exhaled nitric oxide; AUC = area under curve, measured as percent decline in FEV_1_ spanning baseline to 3 h after allergen challenge. The histogram underlying each plot illustrates the distribution of the participants’ OS-GRS. Effect values represent the DBP-attributable difference between the measurement at the given time point and the baseline. The solid line represents the interaction effect, with the p value corresponding to the interaction significance. The shaded region represents 95% confidence intervals. **Figure S3.** PPAR-γ single nucleotide polymorphism rs10865710. Effect modification by genotype G/C or C/C on (A) Blood IgE at 3 and 20 hours post-exposure to CA with allergen challenge (Air+Ag) or DBP with allergen challenge (DBP+Ag), (B) Blood Th2 lymphocytes at 3 and 20 hours post-exposure to CA (Air) or DBP, and (C) BAL Th2 lymphocytes at 24 hours post-exposure to CA (Air) or DBP.

## Data Availability

All relevant data generated or analysed during this study are included in this published article and its supplementary information file. Additional data may be available from the corresponding author on reasonable request.

## Abbreviations

AHR: Airway hyperresponsiveness
AUC: Allergen area under the curve
BAL: Bronchoalveolar lavage
CA: Control air
DBP: Dibutyl phthalate
FEV_1_: Forced expiratory volume in 1 s
FeNO: Fractional exhaled nitric oxide
IgE: Immunoglobulin E
OS-GRS: Oxidative stress genetic risk score
PBMC: Peripheral blood mononuclear cells
PPAR-γ: Peroxisome proliferator-activated receptor gamma
P-GRS: PPAR-γ genetic risk score
SNP: Single nucleotide polymorphism
