# Supplementary material for: Peroxisome proliferator-activated receptor gamma gene variants modify human airway and systemic responses to indoor dibutyl phthalate exposure
Source: Respir Res. 2022 Sep 16;23:248. doi: 10.1186/s12931-022-02174-8 (PMC9482266; doi:10.1186/s12931-022-02174-8)
Supplement: Supplementary file 1 — Additional file 1: Table S1. DNA sequences for PPARG used to perform targeted pyrosequencing. Table S2. The genotypes and GRS are illustrated for rs709158, rs3856806, and rs10865710. Table S3. The weights of each SNP in the construction of P-GRS. Table S4. Oxidative stress-related genes included in the associated OS-GRS and definition of their risk alleles. Table S5. Exposure-by-P-GRS interaction on DBP effects on (A) blood immune cells at 3h, (B) blood immune cells at 20h, and (C) BAL immune cells at 24h. Table S6. Participant characteristics and oxidative stress genetic risk score (OS-GRS). Figure S1. Effect of OS-GRS interaction on DBP effect on IgE and Th2 lymphocytes. Blood allergen-specific IgE at 3 and 20 h (A-B), and BAL allergen-specific IgE at 24 h (C); blood % Th2 lymphocytes at 3 and 20 h (D-E), and BAL % Th2 at 24 h (F) after the allergen challenge post-DBP. The histogram underlying the plot illustrates the distribution of the participants’ OS-GRS. Blood and BAL samples were collected for 16 and 11 participants, respectively. The histogram underlying each plot illustrates the distribution of the participants’ OS-GRS. Effect values represent the DBP-attributable difference between the measurement at the given time point and the baseline. The solid line represents the interaction effect, with the p value corresponding to the interaction significance. The shaded region represents 95% confidence intervals. Figure S2. Exposure-by-OS-GRS interaction on DBP effect on lung function outcomes. Lung function outcomes included: (A) FEV1 (% predicted), ∆20 h; (B) Methacholine challenge (LogPC20), 20 h; (C) FeNO, ∆20 h; (D) Allergen AUC, 3 h. The listed time points refer to the time after the allergen challenge post-DBP or CA exposure. FEV1 = forced expiratory volume in one second; PC20 = concentration leading to 20% fall in FEV1; FeNO = fractional exhaled nitric oxide; AUC = area under curve, measured as percent decline in FEV1 spanning baseline to 3 h after allerge [file 12931_2022_2174_MOESM1_ESM.docx]

**Peroxisome Proliferator-Activated Receptor Gamma Gene variants modify human airway and systemic responses to indoor dibutyl phthalate exposure**

Clarus Leung^1^, Min Hyung Ryu^1^, Anette Kocbach Bølling^2^, Danay Maestre-Batlle^1^, Christopher F. Rider^1^, Anke Hüls^3^, Oscar Urtatiz^4^, Julie L. MacIsaac^4^, Kevin Soon-Keen Lau^1^, David Tse Shen Lin^4^, Michael S. Kobor^4^, Chris Carlsten^1^

**Additional Material**

**Addditional methods**

**Sample processing for blood**

Blood samples were collected pre-exposure, and at 3 and 24 hours post-exposure, using BD Vacutainer® Plus for serum (BD Biosciences, San Jose, CA) and BD Vacutainer® with heparin for plasma. Upon collection, serum samples were left at room temperature for 30 minutes to coagulate. Blood samples were then centrifuged at 1300 relative centrifugal force (rcf) for 10 minutes. Serum and plasma were aliquoted and stored at -80 °C.

**Sample processing for BAL**

At 24 hours post-exposure, some volunteers underwent bronchoscopy. Bronchial wash (BW) was collected by instilling and suctioning 20 mL normal saline (0.9% NaCl) twice with samples pooled. Subsequently, bronchoalveolar lavage (BAL) was collected by instilling and suctioning 50 mL normal saline twice, followed by sample pooling. The same instillation volume was used for all sample collection. BW and BAL were passed through a mesh filter (40 µm pore size) and centrifuged for 15 mins at 475 (rcf). The acellular supernatant of BW and BAL was separated, aliquoted and stored at -80 °C. For this study only BAL was assayed.

**Allergen-specific IgE level**

For allergen-specific IgE level in BAL, two 1ml aliquots of the BAL supernatant were purified with Amicon Ultra-2 3K centrifugal filter units (MilliporeSigma UFC200324). The manufacturer’s protocol was followed with the premixing and desalting/diafiltration steps omitted. Aliquots were thawed, vortexed, and 1.8ml of the sample was added to the device. The centrifugal filter was centrifuged at 3214 rcf for 35 min at 4°C. The filter was inverted and centrifuged at 1000 rcf for 2 min at 4°C to recover the concentrated solute. The allergen-specific IgE level in blood and BAL was then determined using a Phadia 250 system.

**Flow cytometry**

Immunophenotyping analysis of immune cells was done by flow cytometry with analysis completed using FCS Express (v6.04.0034; De Novo Software). Cells were stained with fluorochrome-conjugated antibodies against surface and intracellular proteins following the supplier’s instructions (BD Biosciences, New Jersey, USA), and data collected on a BD FACSCanto II flow cytometer (BD Biosciences, San Jose, CA). Following exclusion of debris, doublets and dead cells, forward and side light scatter characteristics separated monocyte, granulocyte and lymphocyte populations within the CD45^+^ cells. Three different monocyte subsets were identified by their CD14 and CD16 expression level (classical, intermediate, and non-classical monocytes). Neutrophils and eosinophils were identified by their expression of CD24 and (or lack of) CD16 surface marker, within the granulocyte population, respectively. Within the lymphocyte population, CD3^+^CD4^+^ T cell subsets Th1, Th2 and Th17 were identified based on their expression of CD183 and (or lack of) CD196 surface marker. To determine a positive/negative cut-off for the signal, fluorescence minus one (FMO) controls were used. The flow cytometry data were evaluated in terms of: (i) the percentage of cells present in each population identified in the gating strategy, and (ii) the expression of surface markers as mean fluorescence intensity (MFI) for each of these populations.

**FeNO measurement**

Fractional exhaled nitric oxide (FeNO) was measured at before exposures, and at 3 and 20 hours after exposure. NIOX MINO® (Aerocrine, Sweden) was used following the manufacture’s protocol. Two breaths were collected at each timepoint, and measurement was repeated if the two measurements were not within 10% of each other.

**Genotyping**

Peripheral blood mononuclear cells (PBMC) were isolated using SepMate PBMC Isolation tubes and Lymphoprep Density Gradient medium (Stemcell Technologies). The manufacturer’s protocol was followed, except for blood being diluted 1:1 with room temperature RPMI instead of PBS. PBMC DNA was extracted using Qiagen AllPrep DNA/RNA/miRNA universal kits (Qiagen), according to the manufacturer’s recommendations. Pyrosequencing for SNPs were designed using the PyroMark Assay Design 2.0 software (Qiagen). The DNA sequences used to perform targeted pyrosequencing are listed in **Table S2**. Regions containing each SNP to be genotyped were amplified using PCR (HotstarTaq DNA polymerase kit, Qiagen) with 15 min at 95°C, followed by 45 cycles of 95°C for 30s, 58°C for 30s, and 72°C for 30s, and a final 5 min extension step at 72°C, using biotinylated primers. Streptavidin-coated beads were used to isolate DNA, before strand separation (Pyromark Vacuum Prep Workstations, Qiagen) and sequencing by synthesis on a Qiagen Pyromark Q96 MD Pyrosequencer.^1^ The genotype of each participant was determined using Pyromark MD software (Qiagen).

**Additional tables**

**Table 1. DNA sequences for *PPARG* used to perform targeted pyrosequencing.**

| **SNP ID** | **Alleles** | **Strand** | **Co-ordinate** | **Forward Primer (5’>3’)** | **Reverse Primer (5’>3’)** | **Sequencing Primer (5’>3’)** |
| --- | --- | --- | --- | --- | --- | --- |
| rs709158 | A/G | + | 3:12462926 | AGATACGGGGGAGGAAATTCA | *Biotin*-TGCAGCCGTTGTAGTGATATCG | GGAGGAAATTCACTGG |
| rs10865710 | C/G | + | 3:12352948 | *Biotin*-GGCTTTTGGCATTAGATGCTGTTT | CACCACTTAGGCAAGGCAAATG | TAGAATAGCTGTATTTTCCA |
| rs3856806 | C/T | + | 3:12475307 | *Biotin*-TGCCAAGCTGCTCCAGAAA | TCTGTCTCCGTCTTCTTGATCACC | CCTGCAGTAGCTGCAC |

**Table 2.** **The genotypes and GRS are illustrated for rs709158, rs3856806, and rs10865710.** The odds ratios (OR) for each genotype are reported according to Li et al.^2^

|  | **Genotype** | **OR** | **Weight for GRS (ln(OR))** |
| --- | --- | --- | --- |
|  | | | |
| rs709158 | G/G | 0.91 | -0.0944 |
|  | A/G | 0.96 | -0.0472 |
|  | A/A | 1 | 0 |
|  | | | |
| rs10865710 | G/G | 0.53 | -0.6348 |
|  | G/C | 0.98 | -0.3174 |
|  | C/C | 1 | 0 |
|  | | | |
| rs3856806 | T/T | 0.97 | -0.0304 |
|  | C/T | 0.96 | -0.0152 |
|  | C/C | 1 | 0 |

**Table 3. The weights of each SNP in the construction of P-GRS.**

| **ID** | **rs709158** | **rs10865710** | **rs3856806** | **rs1805192** | **P-GRS** |
| --- | --- | --- | --- | --- | --- |
| 1 | -0.0472 | 0 | 0 | 0 | -0.0472 |
| 2 | -0.0472 | 0 | 0 | 0 | -0.0472 |
| 3 | -0.0472 | -0.3174 | 0 | 0 | -0.3646 |
| 4 | -0.0472 | 0 | 0 | 0 | -0.0472 |
| 5 | 0 | 0 | 0 | 0 | 0 |
| 6 | -0.0472 | 0 | 0 | 0 | -0.0472 |
| 7 | -0.0944 | 0 | 0 | 0 | -0.0944 |
| 8 | -0.0472 | -0.3174 | -0.0152 | 0 | -0.3798 |
| 9 | 0 | 0 | 0 | 0 | 0 |
| 10 | -0.0472 | -0.3174 | 0 | 0 | -0.3646 |
| 11 | -0.0472 | 0 | 0 | 0 | -0.0472 |
| 12 | 0 | 0 | 0 | 0 | 0 |
| 13 | 0 | 0 | -0.0152 | 0 | -0.0152 |
| 14 | 0 | 0 | 0 | 0 | 0 |
| 15 | 0 | 0 | -0.0152 | 0 | -0.0152 |
| 16 | -0.0472 | -0.3174 | -0.0152 | 0 | -0.3798 |

SNP rs1805192 did not show any variation in the participants’ genotypes and so effectively did not contribute to P-GRS, which then became a 3-SNP score. The weight of individual SNP was calculated as log odds ratio (score), with a larger magnitude reflecting a greater contributed weight to P-GRS. In the interpretation of P-GRS, a more negative score indicates a lower risk and a more positive number (maximum is 0) indicates higher risk.

**Table 4. Oxidative stress-related genes included in the associated OS-GRS and definition of their risk alleles.**

| Oxidative stress-related genes | Risk allele |
| --- | --- |
| GSTT1 | Null |
| GSTM1 | Null |
| rs6726395 | G |
| rs1800629 | A |
| rs2284367 | G |
| rs4880 | C |
| rs4646903 | G |
| rs1800566 | T |
| rs1051740 | C |
| rs1695 | G |
| rs2917669 | A |
| rs8191438 | G |
| rs28362491 | Deletion |
| rs689452 | C |
| rs2364722 | G |
| rs1138272 | T |

Risk alleles were defined based on prior references.^3–10^ In the absence of a clear definition of risk alleles, the minor allele was used as the risk allele.

**Table 5. Exposure-by-P-GRS interaction on DBP effects on (A) blood immune cells at 3h, (B) blood immune cells at 20h, and (C) BAL immune cells at 24h.**

(A)

| Cell population | Change in DBP effect slope with 1 unit increase of P-GRS | P value for exposure-by-P-GRS interaction |
| --- | --- | --- |
| Granulocytes | 3.05 [-29.52 to 35.62] | 0.84 |
| Neutrophils | 2.35 [-6.57 to 11.27] | 0.58 |
| Eosinophils | -2.29 [-11.54 to 6.97] | 0.60 |
| Monocytes | 4.87 [-2.89 to 12.63] | 0.20 |
| Lymphocytes | -7.66 [-38.12 to 22.79] | 0.60 |
| CD3 T cells | -1 [-26.39 to 24.4] | 0.93 |
| B cells | 4.38 [-4.83 to 13.6] | 0.33 |
| Helper T cells | 0.85 [-6.79 to 8.5] | 0.81 |
| Cytotoxic T cells | -1.36 [-10.29 to 7.57] | 0.75 |
| NK cells | 0.34 [-2.65 to 3.33] | 0.81 |
| Th1 lymphocytes | -3.33 [-24.92 to 18.25] | 0.75 |
| Th2 lymphocytes | -25.2 [-47.72 to -2.69] | **0.03** |
| Th17 lymphocytes | -6.31 [-18.28 to 5.67] | 0.28 |
| Treg lymphocytes | 4.13 [-4.58 to 12.84] | 0.33 |

(B)

| Cell population | Change in DBP effect slope with 1 unit increase of P-GRS | P value for exposure-by-P-GRS interaction |
| --- | --- | --- |
| Granulocytes | -8.92 [-30.04 to 12.21] | 0.38 |
| Neutrophils | -3.64 [-10.48 to 3.20] | 0.27 |
| Eosinophils | 3.66 [-4.61 to 11.93] | 0.36 |
| Monocytes | 6.96 [-1.53 to 15.45] | 0.10 |
| Lymphocytes | -3.5 [-20.75 to 13.75] | 0.67 |
| CD3 T cells | -10.2 [-27.3 to 6.90] | 0.22 |
| B cells | -1.65 [-8.22 to 4.91] | 0.60 |
| Helper T cells | -8.26 [-20.55 to 4.03] | 0.17 |
| Cytotoxic T cells | 0.56 [-6.66 to 7.77] | 0.87 |
| NK cells | 0.85 [-1.74 to 3.44] | 0.49 |
| Th1 lymphocytes | -9.7 [-36.16 to 16.76] | 0.44 |
| Th2 lymphocytes | -39.09 [-57.87 to -20.31] | **0.0005** |
| Th17 lymphocytes | -9.18 [-19.91 to 1.55] | 0.09 |
| Treg lymphocytes | 2.07 [-7.9 to 12.0] | 0.66 |

(C)

| Cell population | Change in DBP effect slope with 1 unit increase of P-GRS | P value for exposure-by-P-GRS interaction |
| --- | --- | --- |
| Total macrophages, % | 2.87 [-46.73 to 52.47] | 0.90 |
| M1, % | -4.84 [-66.29 to 56.61] | 0.86 |
| M2, % | 1.87 [-57.42 to 61.15] | 0.94 |
| Ratio M2/M1 | 8.53 [-21.11 to 38.17] | 0.53 |
| Dendritic cells, % | -11.15 [-39.95 to 17.66] | 0.40 |
| Neutrophils, % | -19.85 [-58.72 to 19.02] | 0.27 |
| Eosinophils, % | 27.55 [-6.52 to 61.62] | 0.10 |
| B cells, % | 0.01 [-11.29 to 11.32] | 0.99 |
| T cells, % | -8.47 [-36.13 to 19.2] | 0.50 |
| NK cells, % | 25.75 [-37.11 to 88.62] | 0.37 |
| Th1 lymphocytes, % | 10.6 [-29.93 to 51.13] | 0.56 |
| Th2 lymphocytes, % | -4.99 [-8.97 to -1.01] | **0.02** |
| Ratio Th1/Th2 | -0.08 [-0.81 to 0.65] | 0.80 |

**Table 6. Participant characteristics and oxidative stress genetic risk score (OS-GRS**)

| Participant | Sex | Baseline FEV_1_ (% predicted) | Baseline methacholine PC_20_ (mg/ml) | OS-GRS |
| --- | --- | --- | --- | --- |
| 1 | F | 76 | 0.3 | 8 |
| 2 | F | 88 | 1.7 | 5 |
| 3 | M | 96 | 2 | 7 |
| 4 | F | 79 | 2.9 | 13 |
| 5 | F | 102 | 6.9 | 6 |
| 6 | F | N/A* | 9.1 | 12 |
| 7 | M | 99 | 14.5 | 12 |
| 8 | M | 93 | 16 | 10 |
| 9 | F | 95 | 47.9 | 11 |
| 10 | F | 91 | 64 | 7 |
| 11 | F | 99 | 121.1 | 8 |
| 12 | M | 94 | 147.1 | 11 |
| 13 | F | 108 | 149.3 | 10 |
| 14 | F | 117 | 301 | 11 |
| 15 | M | 74 | 491.5 | 15 |
| 16 | M | 101 | 1067 | 9 |
| Summary^#^ | M = 6 F = 10 | 94 ± 12^#^ | AHR = 8† | 9.7 ± 2.6^#^ |

Definition of abbreviations: N/A = not available; PC_20_ = provocative concentration of methacholine causing a 20% drop in FEV_1_. OS-GRS was calculated as the sum of the number of risk alleles, maximum theoretical score is 32 for each participant. ^#^ mean ± SD. *Baseline spirometry not available for this participant. Baseline lung function measurements were taken at the time of study recruitment. †Airway hyperresponsiveness (AHR) status defined as provocative concentration of methacholine resulting in 20% drop in FEV1 [PC20] ≤ 16 mg/ml.

**Additional figures**

**Figure 1. Effect of OS-GRS interaction on DBP effect on IgE and Th2 lymphocytes.** Blood allergen-specific IgE at 3 and 20 h (A-B), and BAL allergen-specific IgE at 24 h (C); blood % Th2 lymphocytes at 3 and 20 h (D-E), and BAL % Th2 at 24 h (F) after the allergen challenge post-DBP. The histogram underlying the plot illustrates the distribution of the participants’ OS-GRS. Blood and BAL samples were collected for 16 and 11 participants, respectively. The histogram underlying each plot illustrates the distribution of the participants’ OS-GRS. Effect values represent the DBP-attributable difference between the measurement at the given time point and the baseline. The solid line represents the interaction effect, with the p value corresponding to the interaction significance. The shaded region represents 95% confidence intervals.

**
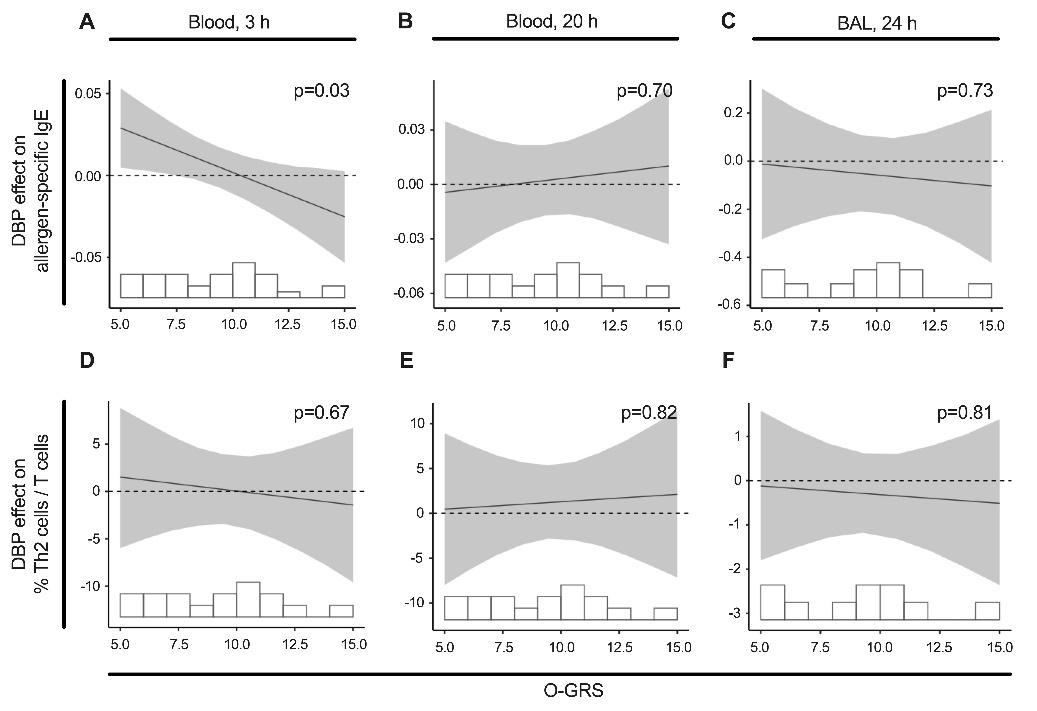
**

**Figure 2. Exposure-by-OS-GRS interaction on DBP effect on lung function outcomes.** Lung function outcomes included: (A) FEV_1_ (% predicted), ∆20 h; (B) Methacholine challenge (LogPC_20_), 20 h; (C) FeNO, ∆20 h; (D) Allergen AUC, 3 h. The listed time points refer to the time after the allergen challenge post-DBP or CA exposure. FEV_1_ = forced expiratory volume in one second; PC_20_ = concentration leading to 20% fall in FEV_1_; FeNO = fractional exhaled nitric oxide; AUC = area under curve, measured as percent decline in FEV_1_ spanning baseline to 3 h after allergen challenge. The histogram underlying each plot illustrates the distribution of the participants’ OS-GRS. Effect values represent the DBP-attributable difference between the measurement at the given time point and the baseline. The solid line represents the interaction effect, with the p value corresponding to the interaction significance. The shaded region represents 95% confidence intervals.


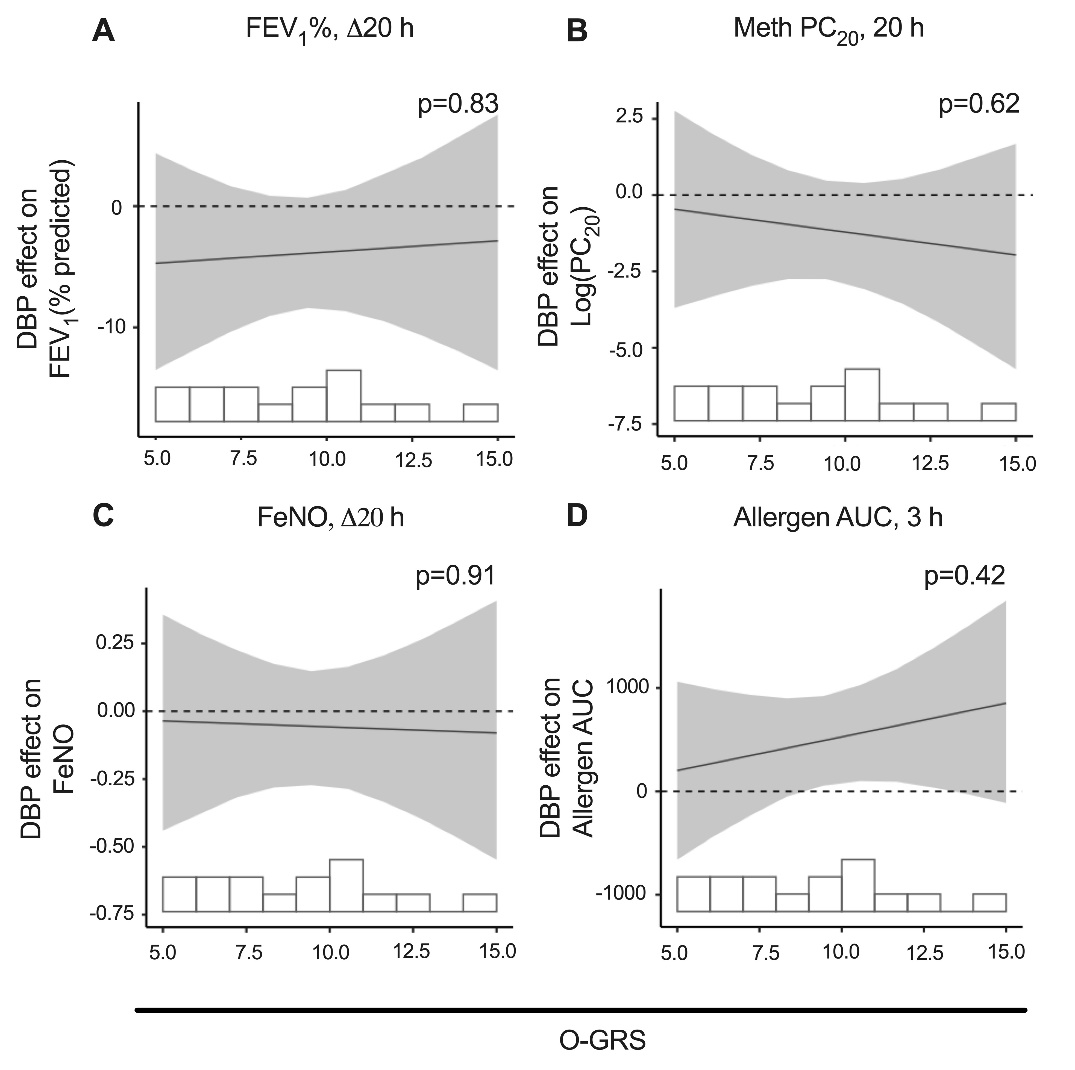


Figure 3. PPAR-γ single nucleotide polymorphism rs10865710. Effect modification by genotype G/C or C/C on (A) Blood IgE at 3 and 20 hours post-exposure to CA with allergen challenge (Air+Ag) or DBP with allergen challenge (DBP+Ag), (B) Blood Th2 lymphocytes at 3 and 20 hours post-exposure to CA (Air) or DBP, and (C) BAL Th2 lymphocytes at 24 hours post-exposure to CA (Air) or DBP.


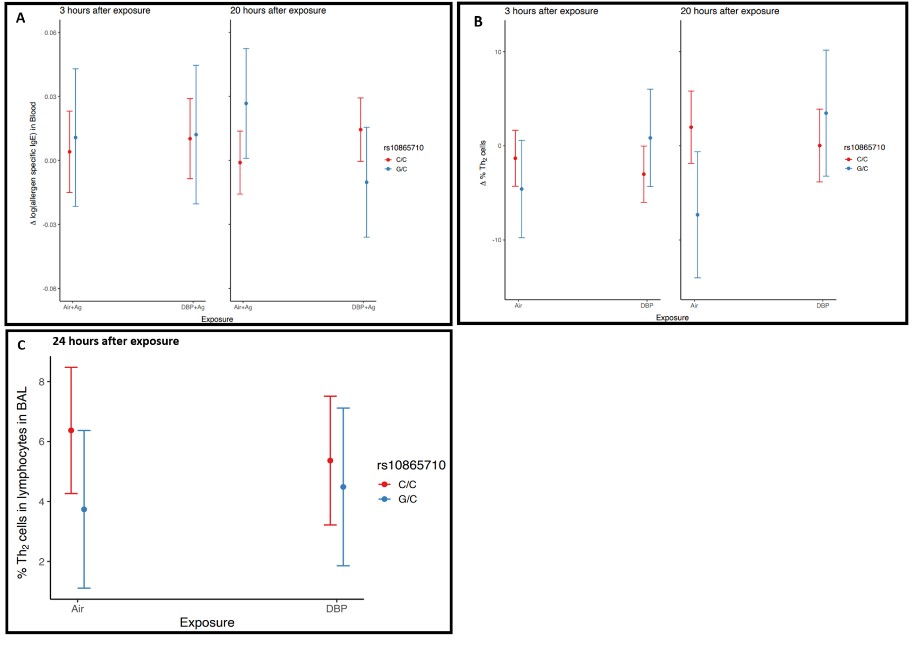


**References**

1. Clifford RL, Jones MJ, MacIsaac JL, et al. Inhalation of diesel exhaust and allergen alters human bronchial epithelium DNA methylation. Journal of Allergy and Clinical Immunology. 2017 Jan;139(1):112–21.

2. Li W, Dai W, Sun J, et al. Association of peroxisome proliferator-activated receptor-gamma gene polymorphisms and gene-gene interaction with asthma risk in a Chinese adults population. Int J Clin Exp Med. 2015;8(10):19346–52.

3. Wooding DJ, Ryu MH, Hüls A, et al. Particle Depletion Does Not Remediate Acute Effects of Traffic-related Air Pollution and Allergen. A Randomized, Double-Blind Crossover Study. Am J Respir Crit Care Med. 2019 Sep 1;200(5):565–74.

4. Bowatte G, Erbas B, Lodge CJ, et al. Traffic-related air pollution exposure over a 5-year period is associated with increased risk of asthma and poor lung function in middle age. Eur Respir J. 2017 Oct;50(4):1602357.

5. Wenzlaff AS, Cote ML, Bock CH, Land SJ, Schwartz AG. GSTM1, GSTT1 and GSTP1 polymorphisms, environmental tobacco smoke exposure and risk of lung cancer among never smokers: a population-based study. Carcinogenesis. 2005 Feb;26(2):395–401.

6. Masuko H, Sakamoto T, Kaneko Y, et al. An interaction between Nrf2 polymorphisms and smoking status affects annual decline in FEV1: a longitudinal retrospective cohort study. BMC Med Genet. 2011 Jul 20;12:97.

7. Aoki T, Hirota T, Tamari M, et al. An association between asthma and TNF-308G/A polymorphism: meta-analysis. J Hum Genet. 2006;51(8):677–85.

8. Mordukhovich I, Wilker E, Suh H, et al. Black carbon exposure, oxidative stress genes, and blood pressure in a repeated-measures study. Environ Health Perspect. 2009 Nov;117(11):1767–72.

9. Canova C, Dunster C, Kelly FJ, et al. PM10-induced hospital admissions for asthma and chronic obstructive pulmonary disease: the modifying effect of individual characteristics. Epidemiology. 2012 Jul;23(4):607–15.

10. MacIntyre EA, Brauer M, Melén E, et al. GSTP1 and TNF Gene variants and associations between air pollution and incident childhood asthma: the traffic, asthma and genetics (TAG) study. Environ Health Perspect. 2014 Apr;122(4):418–24.
